# A replicon-based COVID-19 vaccine candidate delivered by tobacco mosaic virus-like particles

**DOI:** 10.1016/j.vaccine.2025.127063

**Published:** 2025-03-31

**Authors:** Sweta Karan, Patrick Opdensteinen, Yifeng Ma, Jessica Fernanda Affonso De Oliveira, Nicole F. Steinmetz

**Affiliations:** a Aiiso Yufeng Li Family Department of Chemical and Nano Engineering, University of California, San Diego, La Jolla, CA 92093, USA; b Shu and K.C. Chien and Peter Farrell Collaboratory, University of California, San Diego, La Jolla, CA, USA; c Center for Nano-ImmunoEngineering, University of California, San Diego, La Jolla, CA, United States; d Department of Bioengineering, University of California, San Diego, La Jolla, CA, United States; e Department of Radiology, University of California, San Diego, La Jolla, CA, United States; f Institute for Materials Discovery and Design, University of California, San Diego, La Jolla, CA, United States; g Moores Cancer Center, University of California, San Diego, La Jolla, CA, United States; h Center for Engineering in Cancer, Institute of Engineering Medicine, University of California, San Diego, La Jolla, CA, United States

**Keywords:** COVID-19, mRNA vaccine, Tobacco mosaic virus, VLPs

## Abstract

The COVID-19 pandemic highlights the opportunity for mRNA vaccines and their nanotechnology carriers to make an impact as a countermeasure to infectious disease. As alternative to the synthetic lipid nanoparticles or mammalian viruses, we developed a tobacco mosaic virus (TMV)-based mRNA vaccine delivery platform. Specifically, purified coat protein from TMV was used to package a self-amplifying Nodamura replicon expressing the receptor binding domain (RBD) from the Omicron strain of SARS-CoV-2. The replicon construct contains the origin of assembly sequence from the tobacco mosaic virus (TMV) for encapsulation and mRNA stabilization. The nanoparticle vaccine was obtained through *in vitro* assembly using purified TMV coat proteins and *in vitro* transcribed mRNA cassettes. Cell assays confirmed delivery of self-amplifying mRNA vaccine, amplification of the transgene and expression of the target protein, RBD, in mammalian cells. Immunization of mice yielded RBD-specific IgG antibodies that demonstrated neutralization of SARS-CoV-2 using an *in vitro* neutralization assay. The TMV platform nanotechnology does not require ultralow freezers for storage or distribution; and the *in vitro* assembly method provide ‘plug-and-play’ to adapt the vaccine formulation rapidly as new strains or diseases emerge. Finally, opportunity exists to produce and self-assemble the vaccine candidate in plants through molecular farming techniques, which may allow production in the region-for the region and could make a contribution to less resourced areas of the world.

## 1. Introduction

COVID-19 has taught us that rapid development, distribution, and administration of vaccines to the global population is the most effective approach to quell emerging pandemics [1–3]. Vaccine design itself is challenging, but also its manufacture and global distribution; cold chain requirements present logistical and fiscal barriers to the availability of important, life-saving vaccines in resource-poor areas of the world. When designing a vaccine, there are several strategies to choose from and nanotechnology platforms offer great utility in modern vaccine design. The rapid development of a COVID-19 vaccine was possible because the genome and structural information of SARS-CoV-2 was made available in record time [4–6]; and the repurposing of a mature lipid nanoparticle delivery systems enabled rapid clinical development of a COVID-19 vaccine [7]. For fast emerging viral infections and pandemics such as COVID-19, rapid development, and large-scale deployment of vaccines is a critical need.

**Fig. 1.**
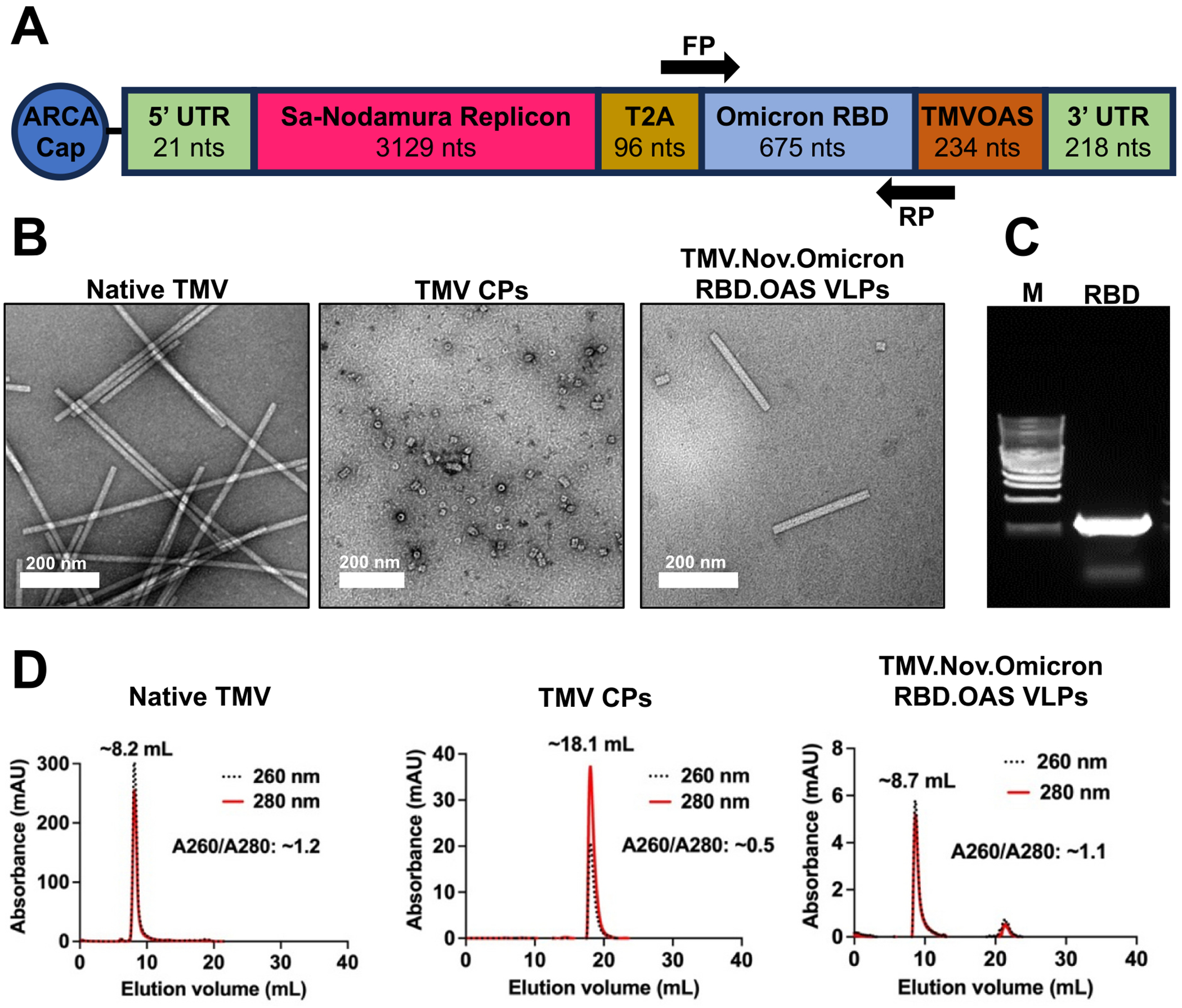
Characterization of TMV.Nov.OmicronRBD.OAS VLPs. **(A)** Schematic representation of Nod.OmicronRBD.OAS mRNA: 5’UTR (21 nts), Nodamura replicon (3129 nts), T2A with linker (96 nts), OmicronRBD (675 nts), TMV OAS (234 nts from 5313 to 5546 in the TMV genome), 3’UTR (218 nts). **(B)** Transmission electron microscopy (TEM) imaging of negatively-stained native TMV, TMV CPs and reassembled TMV.Nod.OmicronRBD.OAS VLPs **(C)** Encapsulation of Nod.OmicronRBD.OAS mRNA into TMV was confirmed by RT-PCR analysis from the RNA isolated from assembled TMV VLPs. The arrow indicates the OmicronRBD specific primer binding site in panel A, with the expected fragment size at 680 bp. **(D)** Size exclusion elution profiles of native TMV, TMV coat proteins (CP), and assembled TMV.Nod.OmicronRBD.OAS VLPs; black dotted and red lines indicate detectors set at 260 nm and 280 nm, respectively. (For interpretation of the references to colour in this figure legend, the reader is referred to the web version of this article.)

While mRNA vaccines offer rapid production and avoid the need for BSL-3 facilities, they are dependent on efficient nanocarriers for delivery. We turned toward the virus-like particles (VLPs) from tobacco mosaic virus (TMV) to package a self-replicating mRNA (making use of the Nodamura virus replicon [8–13]) encoding the SARS-CoV-2 receptor binding domain (RBD) protein (Fig. 1). Nucleic acid delivery using the VLPs from plant viruses and bacteriophages has been demonstrated (reviewed in [14]); and plant virus nanotechnologies including TMV have been applied as mRNA vaccine delivery technology [13,15]. Plant virus nanotechnology is a unique platform offering several distinct advantages: First, native TMV nanoparticles are stable to high temperatures and solvents [16], and therefore do not require refrigeration during storage or distribution. Second, while plant viruses and their VLPs are not infectious toward mammals (thus offering safety), they are highly visible to the immune system – therefore the proposed delivery platform not only serves as a delivery vehicle but also an adjuvant. Their highly organized and repetitive structures act as pathogen-associated molecular patterns (PAMPs), triggering the innate immune system through pattern recognition receptors (PRRs), most commonly toll-like receptors (TLRs) [17]. While the proteinaceous capsids are recognized by TLR2 [18] (and likely other PRRs), RNA-containing VLPs also signal through TLR7/8 [19,20]. These immune-stimulatory properties in combination with their size make them ideal candidates for vaccine delivery to the draining lymph nodes and priming interactions with antigen-presenting cells (APCs) [21].

In this work, we designed a COVID-19 vaccine candidate by making use of a self-amplifying Nodamura virus replicon encoding the RBD domain from SARS-CoV-2 spike protein. The construct was obtained by *in vitro* transcription and then packaged into TMV VLPs through reconstitution using purified coat protein. Nanoparticle vaccines were characterized and transduction efficiency and vaccine efficacy was evaluated in *in vitro* and *in vivo* models demonstrating neutralizing activity against SARS-CoV-2.

## 2. Results

### 2.1. Replicon design and synthesis

For mRNA synthesis, the plasmid constructs pNod.OmicronRBD.OAS and pOmicronRBD were designed by molecular cloning (Fig. 1A and Figure S1). The vector cassettes contained the following features:

- A T7 promoter for *in vitro* transcription of mRNA from the plasmid DNA.
- The vectors were designed with and without the Nodamura replicon (Nod), which was added to increase longevity of the mRNA *in vivo* [8–13].
- The T2A self-cleaving peptide sequence, known for its high “cleavage” efficiency (resulting in a ribosomal skip [22]), was inserted between the Nodamura replicon and OmicronRBD to enable translation of the OmicronRBD protein.
- The antigen was RBD from Omicron strain of SARS-CoV-2 (OmicronRBD).
- Finally, the TMV’s origin of assembly sequence (OAS), a hairpin structure, specifically the 234-nt sequence spanning nucleotides 5313 to 5546 in the TMV genome, was appended to the 3′ end of the mRNA. The OAS facilitates coat protein binding and subsequent nucleation of the cylindrical capsid [23–27].

The mRNA cassette was cloned under the control of the T7 promoter, and linearized plasmids were generated using the *Xba*I restriction enzyme (Figure S2). Hiscribe anti-reverse capping (ARCA)-T7 polymerase was employed for highly efficient transcription of a capped mRNA cassette. The transcription reaction was concluded by “polymerase running-off” from the linearized plasmid template, resulting in the production of capped transcripts Nod.OmicronRBD.OAS and OmicronRBD at the 5′ end. Given the high stability of the Nodamura replicon construct, we chose not to include poly(A) tailing [28].

Analysis of the mRNA transcript concentration, purity, and size was conducted using UV–Visible spectroscopy (not shown) and a bioanalyzer (Figure S2). The absorbance ratio of A260/280 ≈ 2 indicated the purity of the capped mRNA transcripts. This was consistent also with bioanalyzer results confirming pure transcripts with the expected size of −3.7 kb for Nod.OmicronRBD.OAS and – 0.7 kb for the OmicronRBD mRNA (Figure S2).

### 2.2. Packaging the replicons into VLPs

For *in vitro* encapsulation of the transcribed mRNA containing the 234-nts OAS (Fig. 1A) of TMV, we produced TMV coat proteins (CPs) using the established glacial acetic acid [29]. TMV CP was obtained from TMV virions produced in *Nicotiana benthamiana* and established molecular farming and purification methods [30]. Transmission electron microscopy (TEM) imaging shows the typical high aspect ratio rods of native TMV as well as monodispersed and disassembled CP arranged as stacked disks (Fig. 1B). The resulting CPs were then used for packaging the mRNA transcripts at a mass ratio of 20:1 (CP:mRNA). The *in vitro* self-assembled TMV.Nod.OmicronRBD.OAS VLPs revealed typical high aspect ratio nanotubes (length of ~200 nm), which matched the theoretical length of the VLPs which was determined at 206 nm (Fig. 1B). The RNA serves as a ruler defining the length of TMV; native TMV packages a 6.3 kb genome and measures ~300 nm. The Nod.OmicronRBD.OAS replicon measures 4.3 kb and hence results in ~200 nm rods. High resolution size exclusion chromatography (SEC) was used to assess VLPs purity and integrity based on the physicochemical properties. The chromatographic monodisperse peaks observed in our study indicated the purity of native TMV, TMV coat proteins (CP), and reassembled TMV.Nod.OmicronRBD.OAS VLPs. Size exclusion chromatography (SEC) was in good agreement with TEM and showed the typical elution profile of native TMV (~ 8 mL), TMV CPs (~ 18 mL) and reassembled TMV VLPs (~ 8 mL) using a Superose6 Increase column (Fig. 1D). The SEC profiles provide insights into the purity of the plant viral expression vector: This method separates macromolecules (VLPs, CPs, mRNA) based on size, allowing for the separation of intact TMV VLPs from free CPs or unpackaged mRNA as well as other impurities. Due to high molecular weights assembled TMV.Nod.OmicronRBD.OAS VLPs or native TMV eluted at ~8 mL from the Superose6 Increase column. In stark contrast, free CP elutes at ~18 mL, therefore allowing clear differentiation between the states (Fig. 1D). Analyses of the obtained chromatogram from the TMV.Nod.OmicronRBD.OAS VLP samples shows a major peak at 8.7 mL with an A260/280 ratio of ~1.1 indicative of VLPs packaging the mRNA cassette. This peak corresponds to ~90 % of the sample; a 10 % fraction eluted >20 mL which may indicate free CP or mRNA impurities. Overall, the SEC trace is in good agreement with the TEM data and absorbance readings by UV–Vis. Purity of TMV CP free from genomic RNA contamination was determined using UV–Vis, showing an absorbance ratio of A260/A280 = ~0.65 and A280/A250 = ~2; in contrast intact TMV has an absorbance ratio of A260/A280 = 1.2 and A280/A250 = ~1 (not shown). Lastly, to confirm that indeed the target replicon was packaged into the VLPs, total RNA from VLPs was isolated and analyzed by RT-PCR using OmicronRBD gene specific primers – analysis by agarose gel electrophoresis confirmed the expected size and hence packing of Nod.OmicronRBD. OAS mRNA into TMV VLPs (Fig. 1C). Finally, the yield of the assembled TMV.Nod.OmicronRBD.OAS VLPs was determined based on BCA assay or UV absorbance at A280, and recovery yields were ~ 50–60 % of the TMV starting amount.

An optimized purification method was established, and we compared the use of spin filtration using a 100-kDa molecular weight cut-off (MWCO) *vs.* ultracentrifugation over a 30 % (*w*/*v*) sucrose cushion. Purified Nod.OmicronRBD.OAS VLP particles were then analyzed by TEM imaging (Figure S3). When using the spin filters, the dominant structures, accounting for 66 % of particles, were TMV disk aggregates (~18 nm, Figure S3A). Only ~11 % of all particles observed were the target Nod.OmicronRBD.OAS VLPs measuring ~200 nm in length (Figure S3A). The remaining fraction were shorter assemblies or broken particles. The yield of ~200 nm-sized Nod.OmicronRBD.OAS VLPs was significantly increased when using the ultracentrifugation method: the number of disc aggregates was drastically reduced and the frequency of ~200 nm VLPs increased from 11 % to 34 % (Figure S3B). This observation was consistent with literature using ultracentrifugation to eliminate incomplete assemblies of virus particles [31]. We concluded that ultracentrifugation was best suited for purification from assembled Nod.OmicronRBD.OAS VLPs, whereas spin filtration could be used to purify and concentrate TMV disk aggregates before assembly.

### 2.3. Stability of TMV-Nod.OmicronRBD.OAS VLPs

**Fig. 2.**
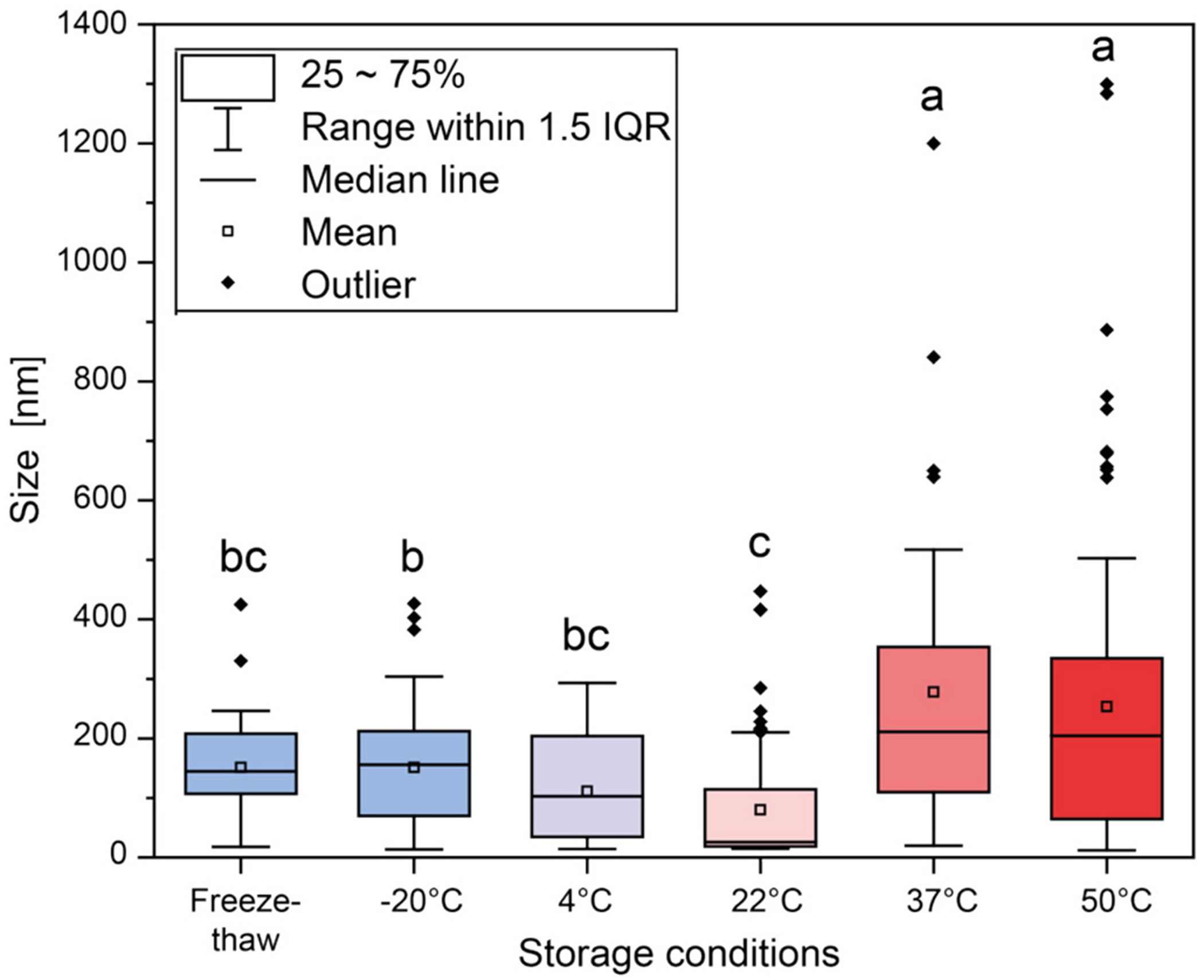
Particle sizes of TMV.Nov.OmicronRBD.OAS VLPs after 1-week at various temperatures. VLP sizes were derived from TEM images using the software ImageJ and evaluated by analysis of variance (ANOVA) using the software OriginPro with a *post hoc* Bonferroni test [38] and a significance level of α = 0.05. Lowercase letters indicate significance groups; conditions that share the same letter were not significant different (*p* > 0.05). IQR – inter quartile range.

Plant viruses are recognized as stable biologics and native TMV remains structurally sound at temperatures up to 50 °C [32]. Therefore, we investigated the stability of the *in vitro* reconstituted TMV-Nod. OmicronRBD.OAS VLPs. Stability was assayed by TEM imaging after 1-week incubation of TMV-Nod.OmicronRBD.OAS VLPs in 10 mM KPO_4_ buffer pH 7 at −20 °C, 4 °C, 22 °C, 37 °C and 50 °C. Frozen TMV-Nod. OmicronRBD.OAS VLPs were also subjected to 5 freeze-thaw cycles (−20 °C, 22 °C) (Figs. 2 and S4). The key findings are: VLPs were stable at 4 °C as well as −20 °C and tolerated repeated freezing and thawing. We note that some particle breakage occurred (slight increase in the faction ~100 nm-sized VLPs, albeit not statistically significant, see Fig. 2) after repeated freeze-thaw cycles (Figure S4A, B), and this is consistent with literature reports [31]. At 4 °C and 22 °C, there is tendency for more TMV disc aggregates (~18 nm) (Figure S4C, D), while at higher temperatures (37 °C and 50 °C), there was a tendency of larger assemblies, formed by free CPs, discs or by end-to-end assembly of VLPs (Figure S4E, F). The tendency for self-assembly as a function of temperature is consistent with other reports [33,34].

Importantly, data indicate that TMV.Nod.OmicronRBD.OAS VLPs are suitable for storage in a fridge (4 °C) or freezer (−20 °C). Significant changes in particle size was observed at elevated temperatures with a size decrease at 22 °C (ANOVA, *p* = 0.0015, *N* = 276, α = 0.05), and increase after storage at 37 °C (ANOVA, *p* < 0.0001, *N* = 126, α = 0.05) or 50 °C (ANOVA, p < 0.0001, *N* = 175, α = 0.05) (Fig. 2). Therefore this vaccine candidate omits the need specialized ultralow freezers (−60 °C to −80 °C) that were required for lipid nanoparticle-based COVID-19 vaccines [35,36]. The tolerance of TMV-Nod.OmicronRBD.OAS toward repeated freeze-thaw cycles is another advantage, considering that cold chain breaches are rather common [37].

### 2.4. Expression of the replicon in vitro

**Fig. 3.**
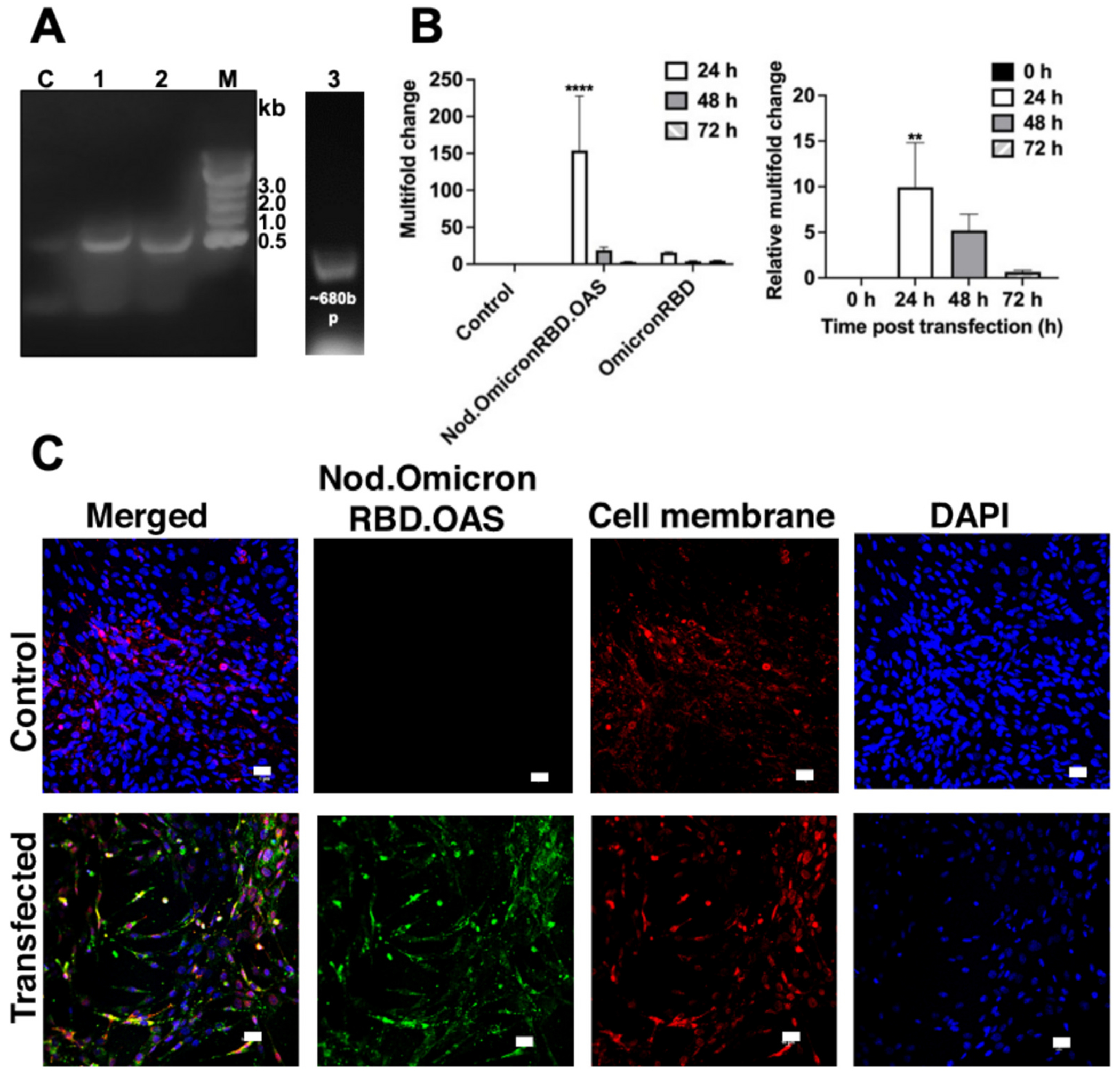
Expression analysis of Nod.OmicronRBD.OAS. **(A)** Transfection of mRNA (500 ng/well) Nod.OmicronRBD.OAS (lane 1) and OmicronRBD (lane 2), or mRNA encapsulated TMV.Nod.OmicronRBD.OAS VLPs (lane 3; 10 μg TMV VLP with ~500 ng Nod.OmicronRBD.OAS mRNA) in BHK-21 cells was confirmed by RT-PCR. Total RNA was isolated from cell lysates after 24 h of transfection. The amplicons of RBD were observed at the expected size ~680 bp on 1 % (*w*/*v*) agarose gel. **(B)** Quantitative analysis of mRNA transcripts by real-time quantitative PCR and multifold change levels of OmicronRBD mRNA were analyzed from the cell lysate collected post-transfection of Nod.OmicronRBD.OAS and OmicronRBD (500 ng/well) at 24 h. OmicronRBD was normalized to the reference gene GAPDH. Also plotted (right panel) is the relative multifold change of Nod.OmicronRBD with respect to the OmicronRBD. Significance level at 24 h was determined by two-way ANOVA using Tukey’s multiple comparison; **** *p* < 0.0001 calculated with respect to the control cells and **p < 0.005 was calculated with respect to the presence of replicon. **(C)** Immunofluorescence imaging of Nod.OmicronRBD.OAS mRNA transfection efficiency in BHK-21 cells using an Omicron-specific anti-SARS-CoV-2 Spike RBD antibody. The scale bar is 10 μm.

To test expression of the transcribed mRNA cassette in mammalian cells, we transfected either free OmicronRBD, free Nod.OmicronRBD. OAS or assembled TMV.Nod.OmicronRBD.OAS VLPs into BHK-21 cells, and observed the expression of RBD. We tested transfection using ‘naked’ mRNA to validate the designed mRNA is functional. RT-PCR analysis was performed from the RNA isolated from the transfected cell lysates indicating the amplicon at the expected size of ~680 bp (Fig. 3A); and this was observed for the ‘naked’ mRNA’ and the TMV-packaged mRNA constructs. Next, we carried out quantitative gene analysis of RBD in either presence or absence of self-amplifying Nodamura replicon. Here we focused on the free mRNA constructs (not VLPs) – to determine the level of amplification as a result of the Nodamura replicon. Indeed significant increase of the OmicronRBD transcript was observed, resulting in a ~ 10-fold increase of Nod.OmicronRBD.OAS *vs.* OmicronRBD 24 h post transfection (Fig. 3B). Nevertheless, even in presence of the replicon, the expression of the transgene was short-lived and negligible at the 72 h time point. While this may offer safety and clearance of the nucleic acid vaccine candidate, the replicon efficiency requires further investigation and potentially optimization. We cannot rule out that differences in the RNAs (size and stability) may also impact their fate in cells.

Finally, expression of the RBD protein in BHK-21 cells was confirmed using Omicron-specific anti-SARS-CoV-2 Spike RBD antibody and confocal imaging. Taken together this data confirms the functionality of the mRNA transcripts (Fig. 3C).

### 2.5. Mice immunization using the TMV.Nod.OmicronRBD.OAS mRNA vaccine candidate

**Fig. 4.**
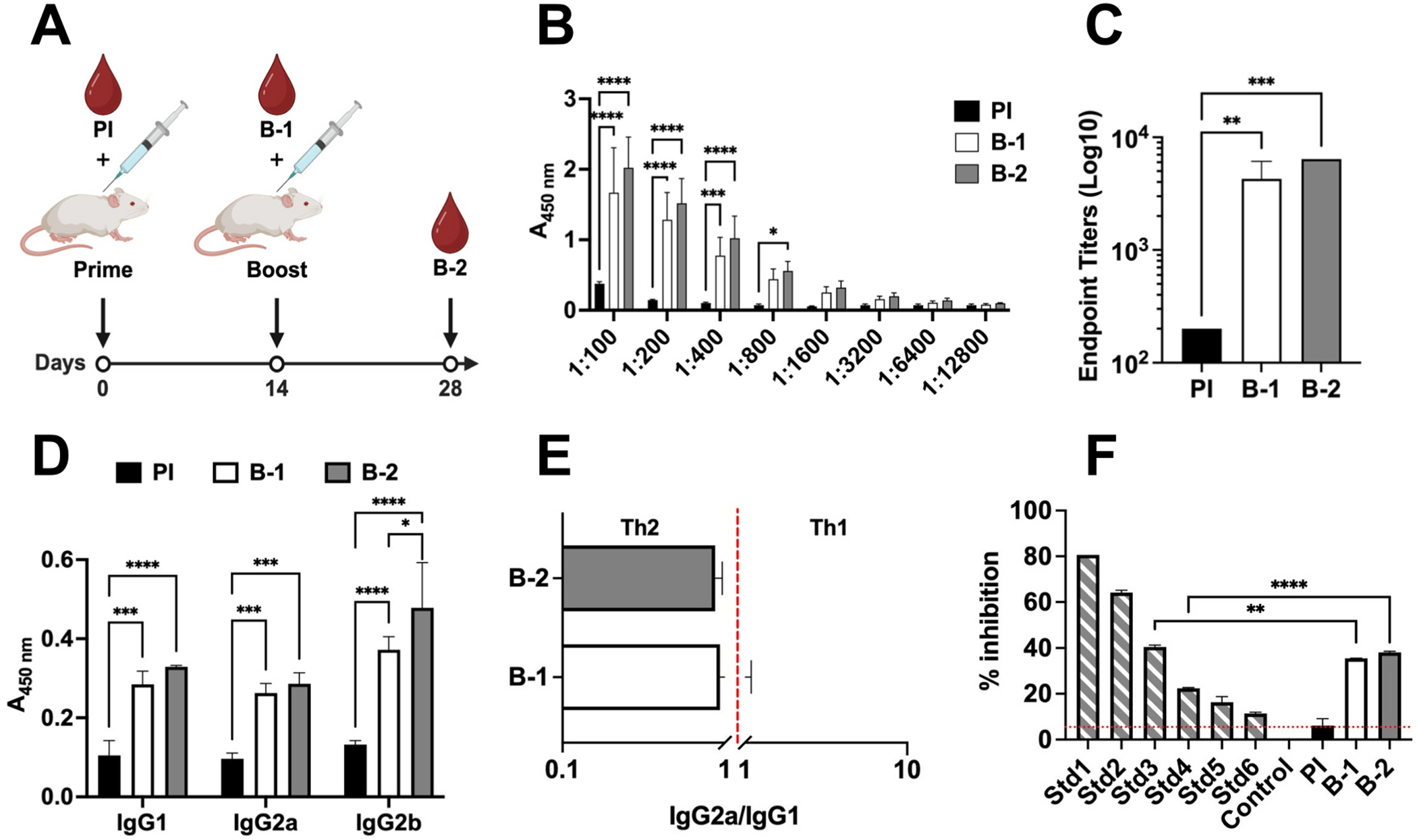
Immunogenicity of TMV.Nod.OmicronRBD.OAS mRNA vaccine. **(A)** Immunization and blood collection schedule scheme using BALB/c mice (n = 5). Scheme made in Biorender. **(B)** Specific anti-OmicronRBD antibodies were elicited post s.c. immunization following a prime-boost schedule [39–41]. Mice received two doses (2 weeks apart) of 100 μg assembled TMV VLPs-based mRNA vaccine and antibody titers against OmicronRBD was analyzed by ELISA, 14- and 28-days post-immunization. **(C)** Endpoint titers showed a ~ 1.5-fold increase in antibodies eliciting post-second immunization. Two-way ANOVA was used to compare between groups using pairwise multiple comparison followed by Tukey’s multiple comparison test. **(D)** IgG subtypes profile (IgG1, IgG2a, or IgG2b) was screened and **(E)** suggested a Th2-biased immune response for our mRNA vaccine candidate. One-way ANOVA followed by Tukey’s multiple comparison test was used to compare between groups. **(F)** Inhibition percentage (%) above cut-off values indicate the presence of neutralizing anti-OmicronRBD antibodies. Std 1–6 is the standard curve – 2-fold serial dilution of a monoclonal control antibody (300 U/mL-4.688 U/mL). One-way ANOVA followed by Tukey’s multiple comparison test was used to compare between groups. Asterisks (****p < 0.0001, ***p < 0.0005, **p < 0.001, *p < 0.05) indicate significant differences between groups.

Effectiveness of the TMV.Nod.OmicronRBD.OAS mRNA vaccine candidate was evaluated in BALB/c mice following a prime-boost immunization schedule (*n* = 5, 100 μg of vaccine candidate, s.c., 14 days apart), as previously reported [39–41]; PBS was used as a placebo. Blood was collected by retro-orbital bleeding prior the first immunization (pre-immunization or PI, Fig. 4A), and then 14- and 28-days post-immunizations (bleed or B-1 and B-2, Fig. 4A). Plasma was separated by centrifugation and screened for anti-OmicronRBD-specific antibodies and IgG subclasses using ELISA. Following the first immunization (prime), the TMV.Nod.OmicronRBD.OAS mRNA vaccine candidate elicited antibodies against Omicron-RBD of SARS-CoV-2 (B.1.1.529) and titers slightly increased after the second immunization (boost; Fig. 4B) reaching an endpoint titer of ~1:4200 (prime) and ~ 1:6400 (boost), respectively (~1.5-fold increase, Fig. 4C). IgG subclasses (IgG1, IgG2a/b) were determined (Fig. 4D) and data suggest an overall balanced Th1/Th2 with a trend toward Th2-bias (IgG2a/IgG1 < 1 ratio) (Fig. 4E).

Finally we assayed whether the antibodies elicited by the TMV.Nod. OmicronRBD.OAS mRNA vaccine candidate were neutralizing. A GenScript cPass SARS-CoV-2 Neutralization Antibody Detection Kit was used – the assay principle is a blocking ELISA that detects IgG that neutralize the interaction between RBD and hACE2 receptor. Pooled plasma (*n* = 5) pre- (PI) and post-immunizations (B-1 and B-2) were used and their neutralizing effect on the OmicronRBD-hACE2 interaction probed. Signal inhibition of >30 % is a positive results – and with ~35 % and ~ 38 % signal inhibition our vaccine candidate indeed elicited neutralization antibodies. Based on the standard curve and 10-fold dilution of the plasma analyzed, the mouse plasma reached a concentration of ~1000 U/mL RBD-specific antibodies (Fig. 4F).

## 3. Discussion

We successfully developed an mRNA vaccine candidate by making use of the TMV coat proteins and *in vitro* packaging of a Nodamura virus replicon-based mRNA cassette enabling RBD expression in cells and more importantly in mice yielding SARS-CoV-2 neutralizing antibodies. More research is need to fully deploy the plant virus-based nanotechnology in vaccine design, *i.e.* benchmarking studies with existing technologies – however the data highlight utility of this platform technology.

The COVID-19 pandemic highlighted the opportunity of platform nanotechnology and power of genetic medicine. Viral and non-viral vector technologies are being developed, but only a small number of platforms is under consideration. Viruses are the most abundant biological entities on the planet with an estimated 10 [31] viruses on Earth – this biodiversity offers tools for biotechnology. Since the discovery of tobacco mosaic virus (TMV) in 1898 [42] and its use for guiding principles of structural biology [16], it has been repurposed for many different applications [16]. A few isolated prior works demonstrated feasibility of plant viral gene delivery: for example, TMV was utilized to deliver mRNA encoding GFP [15]; similar marker gene expression has been demonstrated using cowpea chlorotic mottle virus (CCMV) [10]. CCMV was further engineered to deliver replicons [43,44] enabling amplification and expression of model antigens for vaccine development [13]. These studies underpin the potential of plant virus-based vectors in nucleic acid delivery and in the present work we demonstrate a first step toward the design of a functional vaccine candidate.

Compared to the contemporary viral vectors or synthetic nano-particle systems, plant virus offer advantages: Plant viruses can be considered safer for use in humans compared to their mammalian counterparts, because plant viruses do not replicate in or infect mammals [45]. The system is non-integrating and therefore does not bear the risk insertional mutagenesis [46]. We and others have shown that the plant virus based-delivery system can be administered at doses of up to 100 mg (10 [16] particles) per kg body weight without clinical toxicity [47,48]. The platform demonstrates excellent blood and tissue compatibility [49,50]. Plant viruses are immunogenic and naturally target immune cells – thus providing a means of targeted delivery system with adjuvant properties. Antibody opsonization plays a fundamental role in nanoparticle and plant virus clearance and it aids the targeting immune cells. For example, we demonstrated that prevalence of antibodies against cowpea mosaic virus (CPMV) enhances anti-tumor efficacy of the immunomodulatory CPMV nanoparticle [51].

Further advantages of plant virus-based vectors are the availability of scalable manufacturing processes. Plant viruses and their virus-like particles (VLPs = genome-free versions) can be produced in plants or heterologous expression systems in high yields: manufacture in plants produces yields of up 1–2 g plant virus-based particles per leaf tissue [52]. This is in stark contrast to production of mammalian viral vectors, which are produced in tissue culture which yielding milligrams per 1 L cell culture [53]. The plant virus-based approach also provides advantages compared to synthetic systems: while manufacture of synthetic system is scalable, some systems are not stable in aqueous suspensions [54–57]. Finally, TMV exhibits exceptional stability in various media and across a wide range of temperatures – therefore ultralow freezers are not required for distribution. Innovating vaccine delivery platforms to break cold chain limitations is an efficient solution to safeguard potent vaccination for both wealthy and lower-income countries. Finally, one could further engineer the system to produce and self-assemble TMV-based VLPs laden with the desired replicons through molecular farming involving relatively non-sophisticated technology – possible allowing the production in the region for the region for low-resource areas [58].

Finally, regarding safety, we must also take into consideration that an insect virus, namely the Nodamura virus replicon is utilized in the vaccine design. Given their ability to replicate across a wide variety of hosts, including plants, yeast, and mammalian cells, insect virus replicons have been incorporated in several vaccine designs targeting chikungunya fever, influenza, and zika [59–61]. As the technology matures, detailed safety studies must be conducted – in particular to assay potential genome integration, persistence in host cells, possible generation of virus-like vesicles, and off-target effects through pattern recognition receptor signalling [62].

## 4. Conclusion

In conclusion, we demonstrate proof-of-concept to build a platform nanotechnology making use of the tobacco mosaic virus coat proteins self-assembled to package a replicon-based mRNA cassette – *in vitro* assembly is primed by inclusion of a origin-of-assembly site to facilitate packaging yielding mRNA-laden TMV. Through a combination of *in vitro* and *in vivo* assays we demonstrate effectiveness of mRNA delivery and target antigen expression in mammalian cells and mice. SARS-CoV-2 specific immunoglobulins (IgG) were produced; the immune response was Th1/2 balanced and most important IgGs were neutralizing as demonstrated by blocking of RBD-hACE2 receptor interactions. Because TMV and its coat proteins can be scaled through molecular farming or fermentation and the assembly technology is simple while offering stability, this system holds promise to serve a platform nanotechnology for nucleic acid delivery.

## 5. Methods

### 5.1. Molecular cloning and plasmid construct

The plasmid constructs pT7.Nov.OmicronRBD.OAS and pT7.OmicronRBD expressing ribosome binding domain of the spike protein against the Omicron variant of the SARS-CoV-2 with or without selfamplifying Nodamura replicon (Nod) was cloned into the plasmid pNod.Luc.OAS (kindly provided by Dr. Gelbart’s lab, UCLA) as described earlier in Gitlin et al. 2014. For no replicon construct, cloning was performed in plasmid pET22b(+) using restriction enzyme sites-5’ *Nde*I and 3’*Age*I respectively. These synthetic constructs were obtained (GenScript Co., Piscataway, NJ) and the spike protein region encoding 325–547 amino acids of the ribosome binding domain from the Omicron variant (B.1.1.529) of SARS-CoV-2 was extracted from GenBank with the accession no. LC731729.1. The selected amino acid sequence contains nine cysteine residues indicated to facilitate folding and biological activity of RBD [6,63]. Among these nine cysteine residues, eight form four disulfide bonds (Cys336-Cys361, Cys379-Cys432, Cys391-Cys525) that contribute to the stability and functionality of the RBD protein. The ninth cysteine (Cys480) likely is involved in inter-molecular dimerization but does not impair the stability, folding or functionality of the RBD. Cloning of the coding region encoding for OmicronRBD domain into the plasmids pT7.Nod.OmicronRBD.OAS and pT7.OmicronRBD was confirmed by restriction digestion analysis and sequencing.

### 5.2. mRNA transcript preparation

*In vitro* synthesis of capped mRNA was achieved by transcribing the linearized plasmid DNA template using HiScribe T7 ARCA mRNA Kit (New England Biolabs, Ipswich, MA). The plasmid constructs pT7.Nod. OmicronRBD.OAS and pT7.OmicronRBD was linearized using *Xba*I-a unique restriction enzyme site present at the C-terminus of the plasmid. The linearized plasmids pT7.Nod.OmicronRBD.OAS and pT7.OmicronRBD were transcribed using HiScribe T7 ARCA mRNA Kit (New England Biolabs, Ipswich, MA) following manufactures’s instructions. The transcribed mRNA coding for Nod.OmicronRBD.OAS and Omi- cronRBD were precipitated by using RNase free Lithium chloride solution. The purity and quality of transcribed mRNA were analyzed by UV–Vis spectroscopy, and Agilent 2100 bioanalyzer.

### 5.3. Functional characterization of transcribed mRNA

#### Transfection.

The transcribed Nod.OmicronRBD.OAS and OmicronRBD mRNA were transfected into the mammalian cells by using lipofectamine 2000 reagent (Thermo Fisher Scientific) following manufacturer’s instructions. The Baby Hamster Kidney Fibroblast cells (BHK-21) were seeded into 6-well plates in (80,000; 500 μl) Dulbecco’s Modified Eagle Medium complete medium (Thermo Fisher Scientific) with 10 % (*v*/v) fetal bovine serum (Thermo Fisher Scientific) and 1 % (*w*/*v*) penicillin/streptomycin (Thermo Fisher Scientific). The mRNA (500 ng) and lipofectamine dilution (5 μl) prepared in Opti-MEM^™^ reduced serum medium (Thermo Fisher Scientific) in a ratio of 1:1 followed by incubation for 5 min at room temperature. Then the cationic mRNA-lipids complex formulations were added to the cells and allowed for further incubations at 37 °C for 24–72 h. The transfected cells were analyzed by RT-PCR, quantitative RT-PCR, Confocal imaging.

#### Reverse Transcription PCR (RT-PCR).

To confirm the transfection of Nod.OmicronRBD.OAS and OmicronRBD mRNA, the total RNA was purified from the transfected cells between 24 and 72 h using RNEasy Mini Kit (Qiagen) and the collected cell lysates were homogenised using QIAshredder homogenizer kit (Qiagen, Valencia, CA) as per the manufacturer’s descriptions. SuperScript^™^ IV One-Step RT-PCR (ThermoFischer Scientific) was performed using specific OmicronRBD primers to detect the OmicronRBD from the transfected cells. The reaction mixture comprises the RNA samples isolated from the cells, 2× Platinum^™^ SuperFi^™^ master mix, forward primer (Sequence: CAT ATG CGA GTT CAG CCT AC; 10 μM), reverse primer (Sequence: CTA GAC ACC GGT TCA GAA AT; 10 μM), and SuperScript^™^ IV enzyme in a nuclease-free water. The reaction mixture was incubated for reverse transcription of isolated RNA at 60 °C; 10 min. For the polymerase reaction, initial denaturation at 98 °C; 2 min and then amplification was followed for 40 cycles at 98 °C; 10 s, 60 °C; 10 s and 72 °C; 30 s/kb with the final extension at 72 °C for 5 min. Agarose gel electrophoresis was performed in Tris-acetate-EDTA electrophoresis buffer (1× TAE, pH 8.0) to analyse the amplified PCR product.

#### Quantitative real time PCR (RT-qPCR).

For quantitative analysis of OmicronRBD mRNA with or without the Nodamura replicon in the transfected cells, the RNA collected post transfection at 24–72 h was reverse transcribed by synthesizing complementary DNA using RT [2] first strand kit (Qiagen). SYBR Green-based real time quantification was performed using RT [2]SYBR Green qPCR mastermix (Qiagen) with OmicronRBD and GAPDH-gene specific primers (RBD forward: 5′ - ACG CTT CGC TTC AGT CTA TG-3’,RBD reverse:5′-GTG AAG AAG GGT GCC AGA TTA (GAPDH forward: 5′- GACTTCAACAGTGACTCCCAC-3′, reverse: 5′- TCTGTTGCTGTAGCCAAATTC-3′). Mean CT values and the relative expression of OmicronRBD gene or multifold change of OmicronRBD in the presence or absence of Nodamura replicon were calculated by 2–delta delta CT values. Quantitative real time PCR was performed in triplicate in a CFX96 real time thermal cycler system (Biorad).

### 5.4. Confocal imaging

To visualize the cellular expression of Nod.OmicronRBD.OAS mRNA on the BHK-21 cells, the immunofluorescence staining of the transfected cells and confocal imaging was performed. BHK-21 cells (80,000 cells/500 μL) were seeded on the coverslips (13 mm) in 6 well cell culture plate prior to transfection. The cells were allowed to attain confluency before proceeding for lipofectamine mediated transfection of Nod. OmicronRBD.OAS mRNA (500 ng) as described above. Post 24 h of transfection, the cells were washed with phosphate buffered saline (PBS, pH 7.4) and fixed in 4 % paraformaldehyde in PBS for 10 min at RT. The cell membrane of the transfected cells were stained with wheat germ agglutinin (*WGA*) conjugated with Alexa Fluor 555, (1:1000; Invitrogen^™^W32464) and then permeabilized with 0.2 % Triton^™^ X-100 for 2 min and blocked with 2 % BSA solution prepared in PBS, pH 7.4 buffer for 1 h at RT. Primary antibody labelling with Omicron specific anti-SARS-CoV-2 Spike RBD antibody, mouse IgG1 (1:50; ACROBiosystems, SPD M305) followed secondary antibody labelling using goat antimouse IgG (H&L) - Alexa Fluor^™^ 488 (1:100; Invitrogen^™^A-11001) in blocking solution. After every step, the cells were washed with PBS, pH 7.4 and were mounted onto slides with histology mounting medium fluoroshield containing DAPI (Sigma-Aldrich, St. Louis, MO) The stained cells were visualized using Nikon A1R confocal microscopy and the acquired images were analyzed using Nikon NIS-Elements software.

### 5.5. TMV Coat protein preparation, large scale assembly of TMV and transcribed Nod.OmicronRBD.OAS

TMV coat protein was prepared by using glacial acetic acid degradation method as described earlier [29]. In brief, the native TMV (10 mg in 0.1 M KP buffer, pH 7.4) was treated with 2 volumes of glacial acetic acid on ice for 20 min. The precipitated TMV genomic RNA was removed by centrifugation at 20,000*g* for 20min at 4°C. The supernatant containing TMV coat protein was collected for dialysis. The supernatant was transferred into dialysis tubing using a 6–8 kDa dialysis membrane (Spectra Por S/P 1 Dialysis Membrane; Thermo Fisher Scientific) against MilliQ H_2_O for 48h – 96 h at 4°C, changing water every 12 h. After dialysis, the white precipitate of TMV CP was collected by centrifugation at 20,000 ×*g* for 20min at 4°C. The CP were then resuspended in 75mM sodium phosphate buffer, pH 7.2. *In vitro* self-assembly of transcribed Nod.OmicronRBD.OAS were prepared with a 20:1 (CP:mRNA) mass ratio of TMV CP and mRNA in 75mM sodium phosphate buffer, pH 7.2 and were incubated for 16–18 h at 30 °C [64].

### 5.6. Characterization of assembled TMV VLPs

#### UV–Vis.

Native TMV, TMV CP and assembled TMV.Nod.OmicronRBD.OAS VLPs concentration was measured by UV–Vis spectroscopy (Nanodrop 2000, Thermo Fisher Scientific), using Beer’s Law (TMV, ε_260nm_ = 3 μl.μg^−1^.cm^−1^ and TMV CP, ε_260 nm_ = 1.3μL.μg^−1^. cm^−1^). TMV CP was confirmed by absorbance ratio of A260/A280 = 0.65 and A280/A250 = 2, compared to native or assembled TMV that shows absorbance ratio A260/A_280_ = 1.2.

#### Size exclusion chromatography (SEC).

Purity and integrity of native TMV (1 mg/mL), TMV CP (1 mg/mL) and assembled TMV.Nod.OmicronRBD.OAS (0.2 mg/mL) was assessed using AKTA pure Fast Protein Liquid chromatography system equipped with a Superose6 Increase size exclusion column (GE Healthcare LifeSciences), using a flow rate of 0.5 mL/min, fixing the detectors at 260 nm (nucleic acid) and 280 nm (protein).

#### Transmission Electron Microscopy.

Native TMV, TMV CP and assembled TMV.Nod.OmicronRBD.OAS (0.1–0.5 mg/mL in deionized water (DI H_2_O)) were loaded onto Formvar-carbon coated copper grids (300 mesh; Electron Microscopy Sciences); PELCO easiGlow operating system was used to render more hydrophilic grids. Grids were negative-stained with 2 % (*w*/*v*) uranyl acetate (Agar Scientific) and imaged using Tecnai G2 TF20 High-Resolution electron microscope (FEI, USA). VLP particle sizes were derived from TEM grids using the software ImageJ (https://imagej.net/ij/) and evaluated by analysis of variance (ANOVA) using OriginPro 2024 (OriginLab) with a *post hoc* Bonferroni test [38] and a significance level of α = 0.05. In statistical tests comparing two groups, N refers to the total number of samples from both groups, whereas n refers to the number of samples from a single group.

### 5.7. Mice immunization

Female BALB/c mice (*n* = 5, six- to seven-weeks-old) was obtained from The Jackson Laboratory (Strain #:000651) and animal experiments were conducted in accordance with Institutional Animal Care and Use Committee (IACUC), University of California San Diego. TMV.Nod. OmicronRBD.OAS VLPs (100 μg of VLPs containing ~5 μg Nod.OmicronRBD.OAS mRNA in 1× PBS pH 7.4) were injected in the mice, subcutaneously (s.c.) behind the neck, following a biweekly prime-boost immunization schedule. Blood was collected using lithium-heparin-treated tubes (Thomas Scientific) by retro-orbital bleeding on day 0 (prior immunization, PI), and then on days 14 and 21 postimmunizations (B-1 and B-2). Plasma was separated by centrifugation at 2000 ×*g* for 10 min at 4 °C and stored at - 20 °C until analyzed.

### 5.8. Detection of anti-Omicron-RBD of SARS-CoV-2 (B.1.1.529) specific antibodies in mice plasma

To determine the presence of specific antibodies against Omicron- RBD of SARS-CoV-2 (B.1.1.529), direct enzyme linked immunosorbent assay (ELISA) was performed using 96-well Pierce high binding nickel- activated ELISA plates (Thermo Fisher Scientific). Plates were coated with polyhistidine_10_ tagged Omicron-RBD protein (GenScript Co., 200 ng/100 μL PBS pH 7.4/well) overnight at 4 °C. After every incubation, plates were washed three times with 200 μL/well of PBST (0.05 % (*v*/v) Tween-20 in PBS, pH 7.4). Mice plasma was diluted in two-fold serial dilution (2 % BSA, PBST), added to the plates and incubated for 1 h at room temperature (RT) in a microplate shaker incubator (400 rpm). After washing, plates were incubated with 1:5000 dilution of horse-radish peroxidase (HRP)-labeled goat anti-mouse secondary antibody (BSA 1 % (v/v) in PBST; 100 μL/well; Thermo Fisher Scientific) and incubated for 1 h at room temperature (RT) in a microplate shaker incubator (400 rpm). After a final washing, plates were developed using 1-Step Ultra TMB-ELISA substrate solution (100 μL/well; 3,3′,5,5′-tetramethylbenzidine, Thermo Fisher Scientific) for 2 min at RT, followed by quenching with 50 μL of 2 N sulfuric acid (Spectrum Chemical). Absorbance at 450 nm was measured using an Infinite 200 Pro microplate reader and i-control software (Tecan, Männedorf, Switzerland). Endpoint antibody titers was determined as the reciprocal dilution at which the absorbance value was twice higher than the blank wells without mice plasma (background). Levels of different IgGs subclasses (IgG1, IgG2a and IgG2b) in mice plasma was determined as described above. Plasma samples were diluted at 1:200 (in PBS) followed by incubation for 1 h at room temperature (RT) in a microplate shaker incubator (400 rpm). Secondary HRP-labeled goat anti-mouse antibodies specific for IgG1 (Invitrogen PA174421), IgG2a (Thermo Scientific A-10685), IgG2b (Abcam ab97250) were diluted 1:1000 followed by incubation for 1 h at room temperature (RT) in a microplate shaker incubator (400 rpm). To determine the type of immune responses, the ratio of IgG2a/IgG1 ratio was reported and ratio higher than 1 was considered as a Th1 response.

### 5.9. Omicron SARS-CoV-2 Neutralization assay

cPass SARS-CoV-2 Neutralization Antibody Detection Kit (GenScript Co.) was used to detect the presence of neutralizing antibodies to Omicron SARS-CoV-2 RBD in mice plasma using a blocking Enzyme Linked Immunosorbent Assay (ELISA). The assay was performed according to the manufacturer’s protocol. Briefly, mice plasma, positive and negative controls (provided in the kit) were diluted to 1:10 in sample dilution buffer (Cat No S1–60, GenScript Co.). SARS-CoV-2 (Omicron) neutralizing antibody calibration curve (monoclonal antibodies with neutralization activity to SARS-CoV-2 (Omicron)) was prepared by a 2-fold serial dilution in sample dilution buffer (300 U/mL-4.688 U/mL). Then, calibration curve, diluted mice plasma and controls were mixed with 1:1000 diluted RBD-HRP solution at 37 °C for 30 min; the RBD-HRP neutralization reaction mixtures was then added to the angiotensin converting enzyme-2 (ACE2)- coated assay microtiter plate for another 30 min at 37 °C, to evaluate the interaction of free RBD-HRP with ACE2. Plate was washed four times with wash solution (provided by the manufacturer). For signal development, plate was incubated in dark with TMB solution at 25 °C for 15 min. After adding the stop solution, the absorbance measurement in microtiter plate reader was performed immediately at 450 nm. The signal inhibition (%) was calculated for the neutralizing Omicron SARS-CoV-2 antibodies present in the antisera as mentioned in the formula:

Signal inhibition(%)=(1−ODvalue of sample/OD value of negative control)*100%

The cut-off percentage of signal inhibition ≥30 %, should be considered positive for neutralizing antibodies as claimed by the manufacturer.

## Supplementary Material

1

## Data Availability

Data will be made available on request.

## Appendix A. Supplementary data

Supplementary data to this article can be found online at https://doi.org/10.1016/j.vaccine.2025.127063.
