# Supplementary material for: A replicon-based COVID-19 vaccine candidate delivered by tobacco mosaic virus-like particles
Source: Vaccine. Author manuscript; Available in PMC 2026 Jun 4. (PMC13234663; doi:10.1016/j.vaccine.2025.127063)
Supplement: 1 [file NIHMS2173870-supplement-1.pdf]

Supporting Information

A replicon-based COVID-19 vaccine candidate delivered by tobacco mosaic virus-like particles

Sweta Karan[1,2,3], Patrick Opdensteinen[1,2,3], Yifeng Ma[1,2,3], Jessica Fernanda Affonso De Oliveira[1,2,3], Nicole F. Steinmetz[1-8]*

[1]Aiiso Yufeng Li Family Department of Chemical and Nano Engineering, University of California, San Diego, La Jolla, CA 92093, USA [2]Shu and K.C. Chien and Peter Farrell Collaboratory, University of California, San Diego, La Jolla, CA, USA

[3]Center for Nano-ImmunoEngineering, University of California, San Diego, La Jolla, CA, United States

[4]Department of Bioengineering, University of California, San Diego, La Jolla, CA, United States

[5]Department of Radiology, University of California, San Diego, La Jolla, CA, United States

[6]Institute for Materials Discovery and Design, University of California, San Diego, La Jolla, CA, United States

[7] Moores Cancer Center, University of California, San Diego, La Jolla, CA, United States

[8]Center for Engineering in Cancer, Institute of Engineering Medicine, University of California, San Diego, La Jolla, CA, United States

* Corresponding authors: nsteinmetz@ucsd.edu

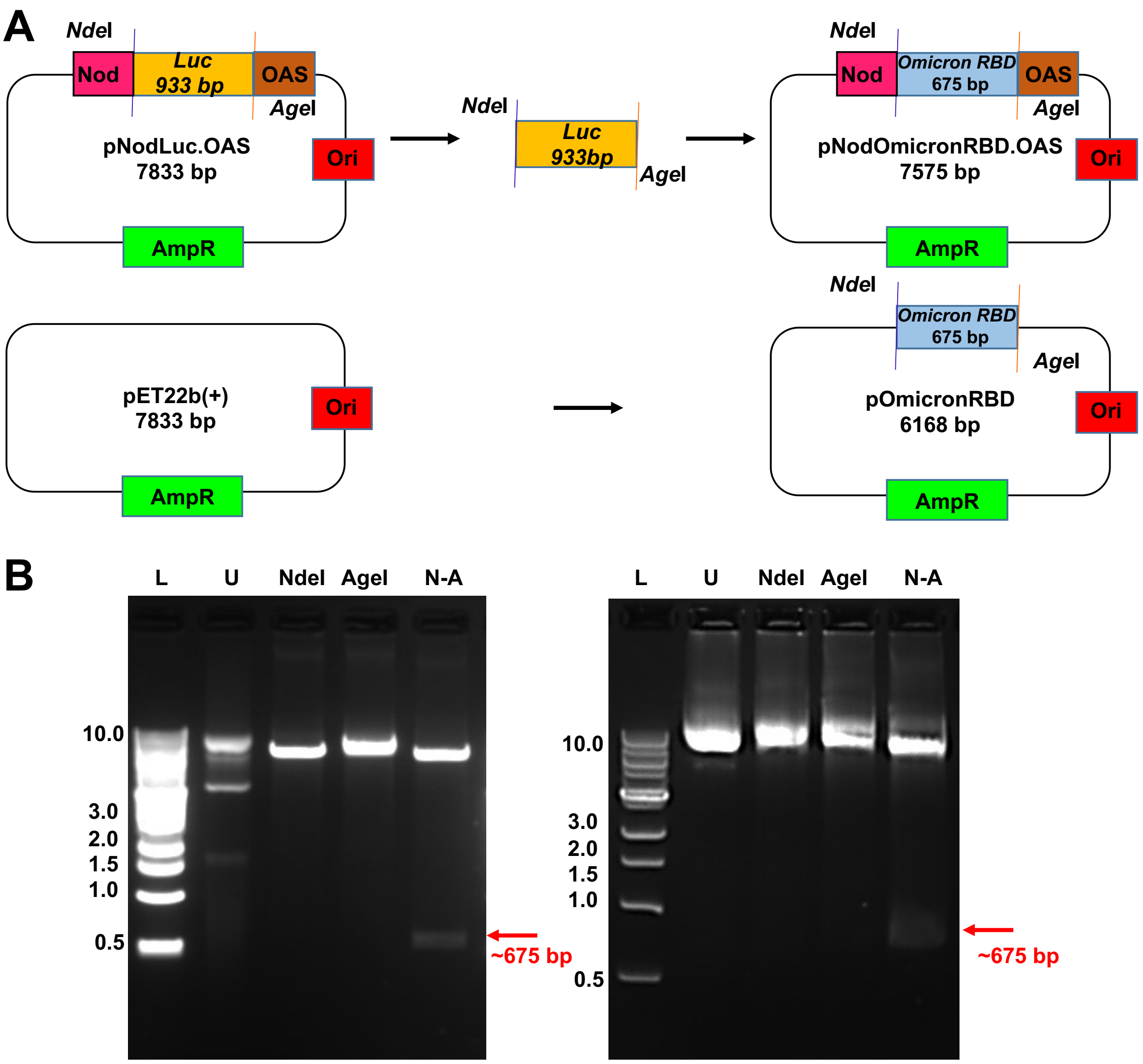

Supplementary Figure 1: Schematic representation of plasmid construct pNod.OmicronRBD.OAS. (A) Subcloning strategy for plasmid pNod.OmicronRBD.OAS and pOmicronRBD. **(B)** Cloning of OmicronRBD gene with replicon and without replicon into the respective vector backbone- pNodLucOAS and pET22b was confirmed by double restriction digestion with *Nde*I and *Age*I on 1% (w/v) agarose gel in 1×TAE (Tris-acetate-EDTA) running buffer. Arrow indicates the release of insert at the expected size ~675 bp.

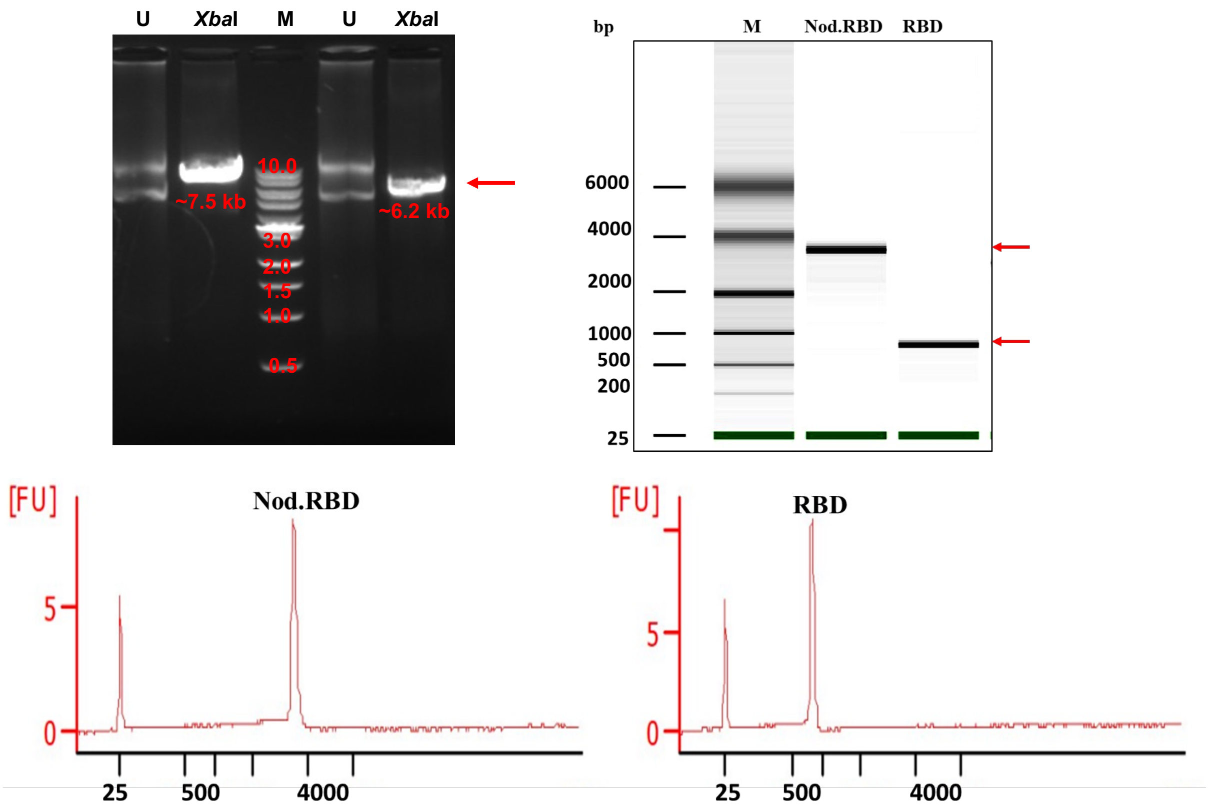

Supplementary Figure 2: *In vitro* transcribed Nod.OmicronRBD.OAS and OmicronRBD mRNA. Analysis of linearized plasmid template on 1 % (w/v) agarose gel in 1×TAE running buffer for in vitro transcription was prepared by single digestion using *Xba*I (Top left panel). Electrophoresis (top right panel) and electropherogram analysis (bottom panel) of *in vitro* transcribed Nod.OmicronRBD.OAS (lane 1, expected size: ~4388 bp) and OmicronRBD mRNA (lane 2: expected size: ~681 bp) on Agilent 2100 bioanalyzer.

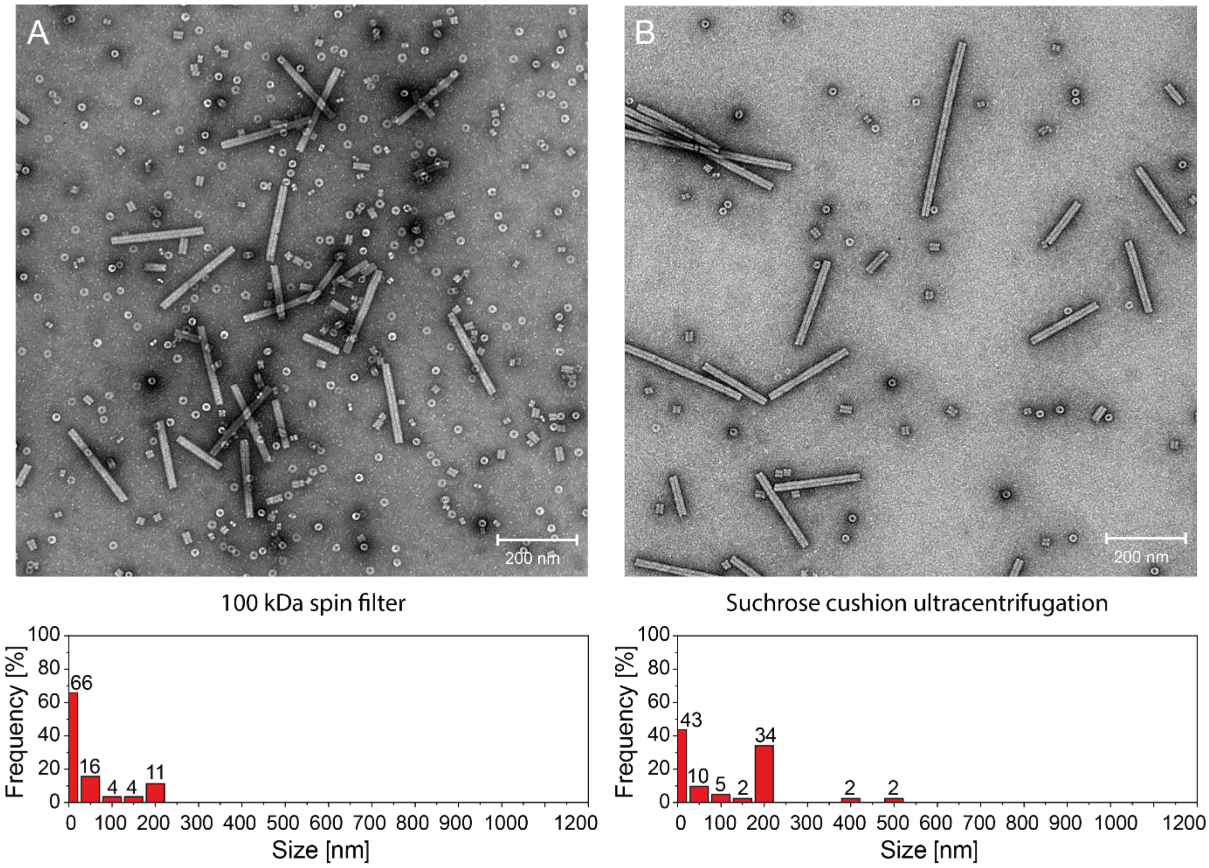

Supplementary Figure 3: Comparison of spin filtration and ultracentrifugation for purification of TMV.Nov.OmicronRBD.OAS VLPs. (A) Transmission electron microscopy (TEM) images of TMV.Nov.OmicronRBD.OAS VLPs and respective particle size distribution after purification with a 100-kDa molecular weight cut-off spin filter. **(B)** TEM images of TMV.Nov.OmicronRBD.OAS VLPs and respective particle size distribution after purification by ultracentrifugation over a 30% (w/v) sucrose cushion. TEM grids were negative-stained with 2% (w/v) uranyl acetate and imaged using a Tecnai G2 TF20 High-Resolution electron microscope. VLP sizes were derived from TEM images using the software ImageJ. The theoretical length of TMV.Nov.OmicronRBD.OAS VLPs is 206 nm.

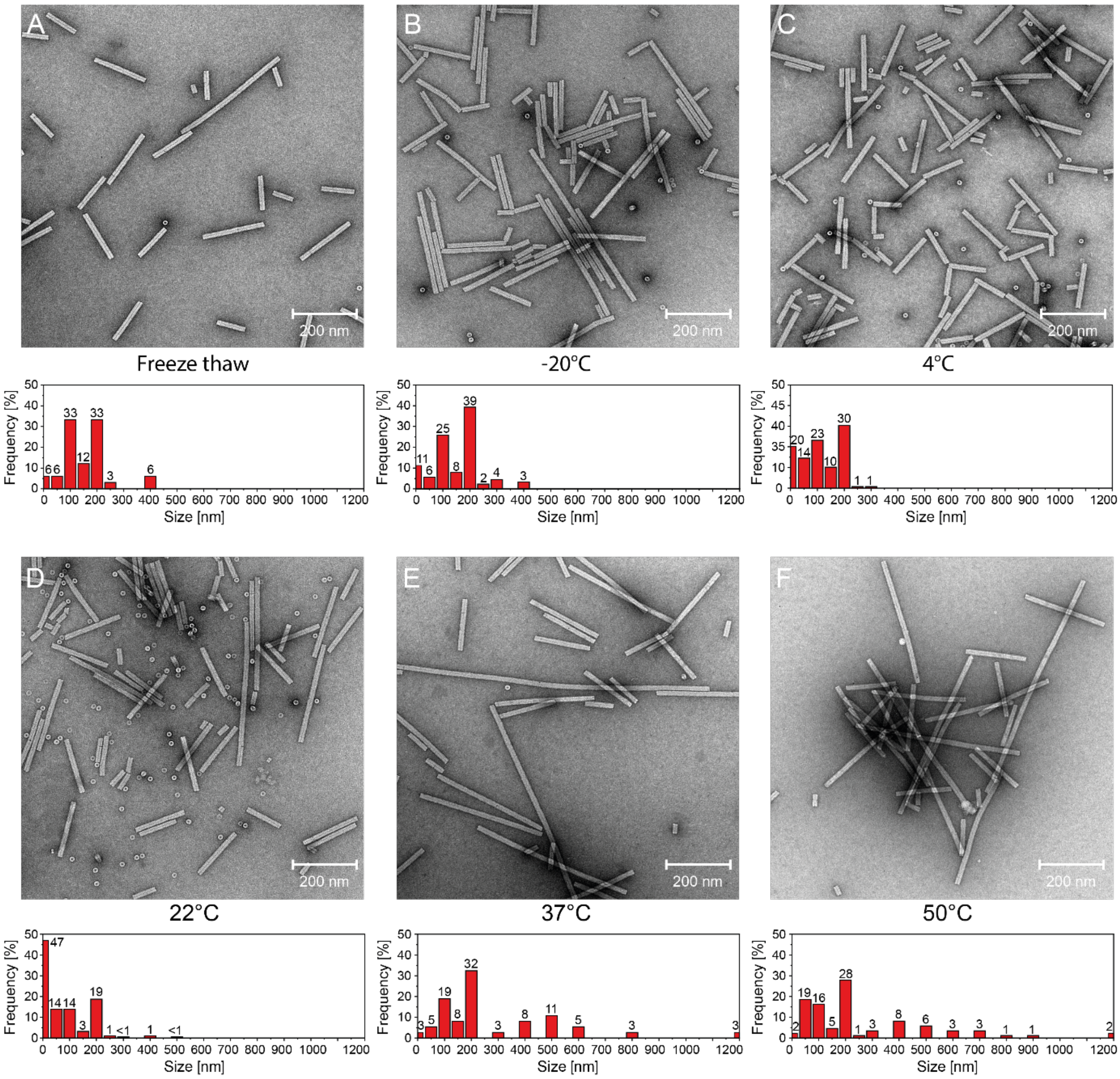

Supplementary Figure 4: Thermal stability of TMV.Nov.OmicronRBD.OAS VLPs. (A-F) TEM images of TMV.Nov.OmicronRBD.OAS VLPs and respective particle size distribution after repeated freezing and thawing **(A)**, storage at -20°C **(B)**, 4°C **(C)**, 22°C **(D)**, 37°C **(E)** and 50°C **(F).** VLPs used for stability studies were purified by ultracentrifugation over a 30% (w/v) sucrose cushion and resuspended in 10 mM KPO_4 buffer. VLPs in **(A)** were subjected to 5 cycles of freezing at -20°C and thawing at 22°C before preparing TEM grids. VLPs in **(B-F)** were stored for 1 week at the indicated temperature before preparing TEM grids. TEM grids were negative-stained with 2% (w/v) uranyl acetate and imaged using a Tecnai G2 TF20 High-Resolution electron microscope. VLP sizes were derived from TEM images using the software ImageJ. The theoretical length of TMV.Nov.OmicronRBD.OAS VLPs is 206 nm.
